# Lived experience of work and long COVID in healthcare staff

**DOI:** 10.1093/occmed/kqad117

**Published:** 2023-12-10

**Authors:** N Torrance, E MacIver, N N Adams, D Skåtun, N Scott, C Kennedy, F Douglas, V Hernandez-Santiago, A Grant

**Affiliations:** School of Nursing, Midwifery & Paramedic Practice, Robert Gordon University, Aberdeen AB10 7QE, UK; School of Nursing, Midwifery & Paramedic Practice, Robert Gordon University, Aberdeen AB10 7QE, UK; School of Nursing, Midwifery & Paramedic Practice, Robert Gordon University, Aberdeen AB10 7QE, UK; Health Economics Research Unit, University of Aberdeen, Aberdeen AB25 2ZD, UK; Institute of Applied Health Sciences, University of Aberdeen, Aberdeen AB25 2ZD, UK; School of Nursing, Midwifery & Paramedic Practice, Robert Gordon University, Aberdeen AB10 7QE, UK; School of Nursing, Midwifery & Paramedic Practice, Robert Gordon University, Aberdeen AB10 7QE, UK; School of Medicine, University of St Andrews, St Andrews KY16 9TF, UK; School of Nursing, Midwifery & Paramedic Practice, Robert Gordon University, Aberdeen AB10 7QE, UK

## Abstract

**Background:**

Healthcare workers (HCWs) had a greater occupational risk of exposure to coronavirus disease 2019 (COVID-19) and reported higher rates of long COVID (LC). This has implications for the provision of health care in already stretched health services.

**Aims:**

This study explored the impact of LC on a range of UK National Health Service (NHS) HCWs, their health and well-being, the effect on work patterns, and occupational support received.

**METHODS:**

Mixed-methods study, online survey and qualitative interviews. Participants self-reporting LC symptoms were recruited through social media and NHS channels. Interviews used maximum variation sampling of 50 HCWs including healthcare professionals, ancillary and administration staff. Thematic analysis was conducted using NVivo software.

**RESULTS:**

A total of 471 HCWs completed the online survey. Multiple LC symptoms were reported, revealing activity limitations for 90%. Two-thirds had taken sick leave, 18% were off-work and 33% reported changes in work duties. There were few differences in work practices by occupational group. Most participants were working but managing complex and dynamic symptoms, with periods of improvement and exacerbation. They engaged in a range of strategies: rest, pacing, planning and prioritizing, with work prioritized over other aspects of life. Symptom improvements were often linked to occupational medicine, managerial, colleague support and flexible workplace adjustments.

**Conclusions:**

LC has a significant impact on the lives of HCWs suffering prolonged symptoms. Due to the variability and dynamic nature of symptoms, workplace support and flexible policies are needed to help retain staff.

## Teaser

Long COVID in healthcare workers is more prevalent than many other occupational groups. Using a mixed-methods design, we examined the health and well-being and work-related factors in healthcare professionals and ancillary staff who self-reported long COVID. Most participants reported multiple symptoms with associated daily activity limitations, and a third reported changes to work duties. We explore the experiences of how they manage their complex and dynamic symptoms while working, including occupational medicine and managerial inputs.

**Key learning points**

**What is already known about this subject**

- During the COVID-19 pandemic, healthcare workers were at greater risk of infection and subsequently reported higher levels of long COVID symptoms, compared to other occupational groups.
- Return to work with long COVID symptoms can be challenging.

**What this study adds**

- This mixed-methods study found that many healthcare workers with long COVID are working but managing complex and dynamic symptoms, with periods of improvement and exacerbation.
- Due to ongoing symptoms of long COVID and poorer HRQL, health workers engaged in a range of strategies to manage, such as rest, pacing, planning and prioritizing, with work prioritized overall.
- Improvement in symptoms was often linked to workplace adjustments (phased return, role, responsibilities, hours, location), and an acceptance they may never get better and/or engage in social activities they had done pre-COVID.

**What impact this may have on practice or policy**

- This study highlights the need for more information on LC in healthcare settings to improve knowledge and promote a better understanding of the condition for colleagues, clinicians, managers and the general population.
- Long COVID is impacting the health and well-being of health service staff and is likely to exacerbate the workforce crisis; people need long-term support and flexible occupational health and human resources policies to enable them to be retained in or reintegrated into their health service role in a useful capacity.
- Occupational health professionals have a crucial role in managing the return to work and flexible workplace adjustments to retain skilled healthcare workers; ease of access to Occupational Health advice for staff based in Primary Health Care is unclear and needs further exploration.

## INTRODUCTION

Healthcare workers (HCWs), many with frontline roles caring for patients with coronavirus disease 2019 (COVID-19), had a greater occupational risk of exposure and developing severe COVID-19 than the general population [1–3]. HCWs also report higher rates of long COVID (LC) compared to other employment sectors, with an estimated 4% of people employed in healthcare reporting symptoms [4].

The term ‘long COVID’ refers to prolonged signs, symptoms and conditions continuing or developing following initial COVID-19 infection that are not explained by alternative diagnosis. Although imprecise, this definition is embodied in current joint National Institute for Health and Care Excellence (NICE) guidelines on the management of long-term effects of COVID-19, where LC is defined as ‘ongoing symptomatic COVID-19’ (symptoms lasting 4–12 weeks) and ‘post-COVID-19 syndrome’ (symptoms beyond 12 weeks) [5]. This aligns with the U.S. Center for Disease Control and Prevention’s definition where LC is broadly defined as signs, symptoms and conditions that continue or develop after initial COVID-19 infection resulting in a ‘wide range of ongoing health problems’ [6], and similar to the World Health Organization’s (WHO) ‘post-COVID-19 condition’ [7].

People experience LC as an illness with a diversity of manifestations and often relapsing–remitting symptoms, uncertain prognosis, and a heavy sense of loss and stigma [8]. Common symptoms include fatigue, breathlessness, chest tightness, cognitive dysfunction and myalgia, with over 200 different symptoms reported [9]. A 2022 systematic review of the global prevalence of LC concluded the prevalence is substantial, symptoms are prolonged and exert pressure on healthcare systems [9]. WHO estimates around 200 million individuals currently experience or have previously experienced LC [10].

The UK National Health Service (NHS) is experiencing unprecedented demand for services, high levels of unfilled staff vacancies and staff burnout [11,12]. An estimated 10 000 NHS staff are absent from work due to LC symptoms [13] with more likely to be working at reduced capacity [14]. This is likely an underestimate as clinical coding for LC is low [15]. Guidance for supporting NHS staff with LC has been published by NHS England [16] and the Society for Occupational Medicine [17], with practical advice on Human Resource (HR) elements, recording of LC-related absence and reasonable adjustments to help support return to work [17]. Retaining NHS workers and reintegrating staff with LC into their NHS role requires more evidence of the impact on occupational factors and changes needed to facilitate the diverse roles in the NHS [17].

This paper is part of a mixed-methods longitudinal study to investigate the lived experience of the longer-term effects of COVID-19 on HCWs, including professional and ancillary staff. The study aimed to explore the impact of symptoms of LC on health and well-being and examine occupational factors around changes in work patterns and support received. This paper presents the results of the cross-sectional data from the first online survey and qualitative interviews.

## METHODS

Participants were eligible to take part if employed in a healthcare setting in NHS Scotland; aged ≥18 years; and self-reported LC signs and symptoms that continued or developed after acute COVID-19. This included both ongoing symptomatic COVID-19 (from 4 to 12 weeks) and post-COVID-19 symptoms (12 weeks or more) [5]. A positive test was not a requirement for participation as these were not available to all HCWs early in the pandemic. A mixed-methods design was used (survey, interviews) with data collected between June 2021 and July 2022.

Participants were recruited via online LC support groups and social media (Twitter, Facebook). An expert advisory group (some with lived experience) assisted with raising awareness. NHS Boards’ communications teams were asked to circulate information about the study to staff.

The online questionnaire included questions about LC symptoms, health and experiences around working in the NHS. The symptoms checklist was generated from published literature and national guidelines [5,18]. Other questionnaire items included socio-demographic characteristics, work setting and prior long-term health conditions. Health-related quality of life (HRQL) was assessed using the Medical Outcomes Short-Form-12 (SF-12) [19], and EuroQol five-dimensional five-level questionnaire (EQ-5D-5L) [20]. Information on depression and anxiety symptoms was collected using the Patient Health Questionnaire for Depression and Anxiety-4 (PHQ-4) [21]. Fatigue was assessed using the Promis SF-V1-4A Measure of Fatigue [22]. Questions about work, in relation to LC symptoms, asked about changes to duties, contracted hours, NHS employer supports and future employment intentions. Data analyses were conducted in IBM SPSS Statistics v28 and Stata v17. Descriptive statistics are reported.

In-depth interviews were conducted with 50 HCWs. Participants were purposively sampled using maximum variation sampling to ensure a range of occupations, socio-demographic characteristics and severity of LC. Interviews (lasting 30–120 minutes) were conducted (by three researchers E.M., N.A. and A.G.) and recorded using MS Teams or telephone. Framework analysis was conducted using NVivo software [23].

Ethical approval was obtained from Robert Gordon University, School Ethical Review Panel (Ref:21-04). Study-wide generic review and NHS Research & Development management approvals were obtained from all NHS Scotland Health Boards (IRAS: 298496).

## RESULTS

A total of 471 HCWs completed the online survey. The majority were female (*n* = 463, 93%), 48% were nursing staff (*n* = 226), over half were in the 36–55 year age category (*n* = 277, 59%) and 54% worked full time (*n* = 244) (Table 1). Two hundred ninety-eight (64%) reported a positive test for COVID-19 when first having symptoms and 68 (14%) had been admitted to hospital.

**Table 1.** Characteristics of the study population (*n* = 471)

	*n* (%)
Age	
Under 25 years	19 (4)
26–35 years	80 (17)
36–-45 years	124 (26)
46–55 years	153 (33)
>56 years	93 (20)
Sex	
Female	431 (93)
Male	35 (8)
Prefer not to say	5 (3)
Ethnic group	
White	456 (98)
Non-White	9 (2)
Occupational group	
Nurse and midwives	226 (48)
Doctor	37 (8)
Allied Health Professional	52 (11)
Ancillary	51 (11)
Administrative	79 (17)
Other	26 (6)
Work setting	
Hospital	282 (60)
Community	129 (27)
Other location	59 (13)
Working time	
Full time	244 (54)
Part time (21–37.5 hours/week)	176 (39)
Part time (<21 hours/week)	33 (7)
No longer working (unable, retired, N/K)	18 (4)
Smoking status	
Non-smoker	341 (74)
Current smoker	21 (5)
Previous smoker	102 (22)
No. of pre-existing health conditions	
None	259 (55)
1	130 (28)
≥2	82 (17)
Duration of LC symptoms	
1–3 months	41 (9)
3–6 months	61 (13)
6–12 months	141 (30)
More than 12 months	227 (48)
Frequency of symptoms	
All of the time	148 (32)
Most of the time	205 (44)
Some of the time	93 (20)
Occasionally	23 (5)
Limitations carrying out day-to-day activities	
A lot	241 (51)
A little	189 (40)
Not at all	39 (8)

The mean number of symptoms reported was 9.5 (standard deviation [SD] 5.2), including fatigue (88%), ‘brain fog’ (79%), breathlessness (69%), sleep disturbance (54%) and heart palpitations (48%) (full list of symptoms reported in the Table 1, available as Supplementary data at *Occupational Medicine* Online. Thirty-one per cent of respondents (*n* = 148) experienced LC symptoms ‘all of the time’, and 64% (*n* = 298) ‘most or some of the time’. LC symptoms adversely affected the day-to-day activities of over 90% of participants, with 51% reporting these were ‘limited a lot’.

HRQL scores, mental well-being and fatigue data are shown in Table 2. Mean physical and mental component scores were lower than general population averages [24]. EQ-5D-5L mean Visual Analogue Scale (VAS) score was 61.5 (SD 19.5), and health utility index score was 0.66 (SD 0.22). Overall, moderate/severe psychological stress was reported by 28% (*n* = 133). The mean PROMIS fatigue *T*-score was 62.6 (SD 9.7) indicating greater fatigue (than the mean standardized US population score of 50 [SD 10]) [22].

**Table 2.** Health-related quality of life, mental well-being and fatigue

In general health now	*n* (%)
Excellent	13 (3)
Very good	51 (11)
Good	137 (29)
Fair	177 (38)
Poor	93 (20)
Health-Related Quality of Life (SF-12)	Mean (SD)
Physical Component Score	38.0 (6.7)
Mental Component Score	41.9 (5.2)
EQ-5D-5L-VAS	61.5 (19.5)
EQ-5D-5L index	0.66 (0.22)
Psychological Stress (PHQ-4)	*n* (%)
Normal	190 (41)
Mild	146 (31)
Moderate	89 (19)
Severe	44 (9)
PHQ—Anxiety	
≤3	365 (78)
>3	104 (22)
PHQ—Depression	
≤3	376 (80)
>3	93 (20)
Promis Fatigue SF 4a, mean (SD)	62.6 (9.7)

SF-12 Physical and Mental Component Score ranges from 0 to 100, with higher scores indicating better physical and mental health functioning. EQ-5D-5L Visual Analogue Scale (VAS) records self-rated health with minimum score of 0 (worst health) and maximum score of 100 (the best health you can imagine). EQ-5D health utility index scores were calculated with values are anchored at 1 (full health) and 0 (less than 0 is a state ‘worse than death’). PHQ-4: Four-Item Patient Health Questionnaire for Anxiety and Depression. Minimum score of 0. Maximum score of 12. Scores are rated as normal (0–2), mild (3–5), moderate (6–8) and severe (9–12). PHQ-4 Anxiety component. Total score >3 suggests anxiety. PHQ-4 Depression component. Total score >3 suggests depression. Promis SF-V1-4A. Measure of Fatigue. A higher *T*-score represents more fatigue and mean standardized US population score is 50 (SD 10).

Almost two-thirds (65%, *n* = 306) had taken sick leave and 48% (216/457) had been issued a medical certificate because of LC symptoms. Overall, 18% (*n* = 79) of participants were unable to work at the time of the survey because of LC-related health problems. For those working, a third (*n* = 148, 33%) reported changes to work duties. Most (*n* = 329, 80%) had not changed work hours, *n* = 71 (17%) had reduced their hours (Table 3). Overall, 10% (*n* = 44) were considering leaving NHS employment in the next year, with a further 24% (*n* = 104) ‘unsure’. Less than half (49%, *n* = 217) were satisfied with support received from NHS employers regarding LC symptoms (Table 4).

**Table 3.** Compared to before you had long COVID, have your contracted work hours changed (if applicable)? *n*, %

	No change	Reduced	Increased	Total
Nurses and midwives	156 (80)	36 (18)	4 (2)	196 (48)
Doctors	21 (66)	6 (19)	5 (16)	32 (8)
Ancillary	34 (79)	7 (16)	2 (5)	43 (11)
Allied Health Professionals	34 (77)	10 (23)	0	44 (11)
Admin	62 (87)	9 (13)	0	71 (17)
Other	22 (88)	3 (12)	0	25 (6)
Total	329 (80)	71 (17)	11 (3)	411

% shown within occupational group.

**Table 4.** Overall, how satisfied are you with the support you have received from your employer in relation to your long COVID symptoms? n, %

	Extremely satisfied	Somewhat satisfied	Neither satisfied nor dissatisfied	Somewhat dissatisfied	Extremely dissatisfied	Total
Nurses and midwives	43 (20)	55 (26)	65 (30)	27 (13)	26 (12)	216 (48)
Doctors	7 (21)	12 (35)	7 (21)	4 (12)	4 (12)	34 (8)
Ancillary	17 (35)	8 (16)	15 (31)	6 (12)	3 (6)	49 (11)
Allied Health Professionals	15 (30)	14 (28)	11 (22)	8 (16)	2 (4)	50 (11)
Admin	22 (30)	11 (15)	29 (40)	7 (10)	4 (6)	73 (16)
Other	9 (36)	4 (15)	9 (35)	2 (8)	1 (4)	25 (6)
Total	113 (26)	104 (23)	136 (30)	54 (12)	40 (9)	447

% shown within occupational group.

Characteristics of the 50 participants interviewed are shown in Table 5: at the time, 37 were working in the NHS and 13 off-work (including 1 who had taken early retirement), due to LC. Of those working, there were 12 nurses, 7 medics, 9 AHPs and 9 ancillary staff: predominantly middle to older aged (36–65 years). Those off-work were younger (26–45 years) and 9/13 were medics or nurses.

**Table 5.** Socio-demographic characteristics and workplace context of participants in qualitative interviews

	Ancillary admin (*n* = 10)	AHP (*n* = 10)	Medic (*n* = 10)	Nurse (*n* = 20)	Total (*n* = 50)
BAME*	1	2	2	1	6
Age ≤25 years	0	0	0	2	2
26-35 years	1	2	1	3	7
36-45 years	4	3	5	1	13
46-55 years	2	3	3	11	19
56-65 years	3	2	1	2	8
66+ years	0	0	0	1	1
Male	2	1	3	2	8
Female	8	9	7	18	42
Primary care	1	1	4	6	12
Secondary care	9	9	6	14	38
Total	10	10	10	20	50

Key to their ability to get back or remain at work were: nature and severity of symptoms; availability of workplace supports (especially flexibility); specific demands and expectations of roles; and own self-management/coping strategies.

Fatigue, breathlessness, brain fog and pain were the most frequently reported. Anxiety and depression were commonly discussed, particularly amongst those off-work. LC symptoms had often been attributed to mental, rather than physical causes by GPs and other healthcare professionals, often frustrating individuals that physical causes were overlooked. Some were anxious or depressed dealing with their illness and the impact on their lives.

Whilst those with the least severe symptoms were working, this did not necessarily indicate recovery or near recovery. The majority had some symptoms, and for some, severe and debilitating, affecting their ability to function at work.

The majority working had prolonged and/or repeated periods off-work. Only seven had never taken time off (or used annual or parental leave to recover), because they felt able to undertake their job despite symptoms or unable to take time off due to demands of their role (Quote 1, Table 6). Several queried whether they would have had quicker or better recovery if they had taken time off.

**Table 6.** Qualitative illustrative comments

Quote number and description	Illustrative quote
1. Unable to take time off due to inherent pressures.	*‘… if you’re not there, your colleagues are having to pick up because there is no slack, there’s no capacity, we don’t have any kind of bank or anything like that, and so you keep going’.* (Medic, Secondary)
2. Difficulties managing job due to cognitive symptoms and high-functioning role.	*‘It’s not the kind of job where you can just be, it’s, it’s not an ordinary job. As a GP … you’re dealing with complexities all the time, so you need to be at the top of your game very sharp all the time. You can’t afford to just be struggling or slowed..’.* (Medic, Primary)
3. Back at work with changed responsibilities and flexibility.	*‘I started back … just a few hours a week over two days … , I was given a rotation … where I could do a lot of work on the computer and just sit, basically, and do office work … So that was great, and my colleagues there were fantastic, very understanding, and were perfectly happy for me to, … (be) given a lot of flexibility … And obviously, if I have a really bad day where I’m very dizzy or feeling unwell … there’s always a seated option’.* (AHP, Secondary)
4. Going back to work too soon detrimental	*‘… well, it got a little bit better and … I went back to work and that made me much worse and I was back in hospital. And then since then I’ve not been at work’.* (Medic, Secondary)
5. Difficulties or delays accessing appropriate and timely support from OH	*‘… my direct line manager’s been great … but OH doctor, she herself has been great, very understanding, and her recommendations have been good and, again, it’s collaborative. But there has been so much delay, in terms of getting to me when I needed it. So, when there were practical, quick decisions that could’ve been made, for me to stay at work, let’s say, you know, reduce hours, something like that, we couldn’t move ahead until we had that report. And the delay was like a couple of months. By then, I got worse’.* (AHP, Secondary)
6. Minimal help from OH in addressing symptoms.:	*‘… I’ve not found OH really helpful. I mean, they’re helpful in one way, cause they agree that I’m not fit to work and tell my management that, which is good to have them on side but they do have resources, like occupational therapy and physiotherapy and things … I don’t know, they don’t seem to think that I need anything from them’.* (Medic, Secondary)
7. Little support from OH until ready to return to work.	*‘So, OH, they’ve helped but they feel they’re at the end, they can’t do anymore unless I’m actually going to be able to be fit to go back to work. They don’t think that I’ll be fit to go back to work for a fair bit of time. So, they’re not wanting to be involved until they think that I’m going …’* (Nurse, Secondary)
8. OH helpful in supporting reasonable adjustments to facilitate return to work.	*‘… they [line-manager] also put me in touch with OH and I had a phone call with them, and I explained everything that was going on, and they were really supportive, actually, they said, when I do think that I can, they give me milestones, they said, if I can walk to the shops and home and not need to rest, then I can look at coming back and they’ll put stuff like, taking out like personal care with patients to maybe make the, the job less strenuous’.* (Nurse, Secondary)
9. Resentment about lack of support—likely contracted COVID-19 at work.	*‘… bit of annoyance at that the fact that the most likely place that I got it, got this was from work … . I guess, I don’t know, the way that they then, you don’t get quite as much support as you probably need’.* (Nurse, Secondary)
10. Uncertainty and anxiety around LC special payment.	*‘… at the start of the pandemic, the Scottish Government said that, you know, those of us working in healthcare wouldn’t lose out financially. So they still pay me for my, my hospital role. How long they’ll do that for I don’t know …’* (Medic, Secondary)
11. Ineligibility to LC special leave.	*‘… I’m struggling at the minute … being off work, to prove I had COVID away back March. Because the impacts the leave that I’m entitled to, at the minute I’m on sick leave, but if I could prove I had COVID I’d be on special leave, which wouldn’t accrue sick leave … And I don’t think there’s any way I can do that now. Yeah, well, financially that will become an issue … because I’ve been on sick leave, I’m now gonna go to half pay’.* (Nurse, Secondary)

Those off-work reported the most severe and debilitating symptoms, most had at least one attempt at returning, and some had repeated attempts. Fatigue and cognitive issues (poor concentration, brain fog) most significantly inhibited return to work. Many medics (and a few nurses and AHPs) discussed challenges managing their high-level and complex work (Quote 2, Table 6). Nurses reflected on their role as ‘fixer’ or ‘carer’ and the challenges LC had presented in maintaining this at work. A fifth of participants discussed how their confidence at work was affected because of changes to cognitive and mental abilities. Others described physical or mobility symptoms as creating difficulties. When they returned to work, it was common for duties to be modified, for example, desk-based tasks (Quote 3, Table 6), changed working patterns to allow rest days. There was often recognition for increased pressure on colleagues ‘picking up the slack’.

Workplace support was fundamental to returning and remaining at work and was discussed at multi-levels: colleagues and team; line management, organizational (NHS) and Occupational Health (OH). The importance of being ‘believed’ about their LC, particularly amongst colleagues and line managers, emerged.

Over two-thirds of those working identified some positive experiences of understanding and support from line manager or colleagues. Around a third discussed beneficial factors: change in role/responsibilities; phased return; reduced hours and/or hybrid working.

Conversely, for those who felt unsupported at work, experiences varied including where there were changes in line management, or where their LC was undisclosed.

Some off-work reported a lack of support and/or communications from their line manager, team and/or OH. Participants described workplaces that were over-stretched, exacerbated by additional pandemic pressures and felt guilty at being off or pressured to return to work, which led to an exacerbation of symptoms and necessitated further time off (Quote 4, Table 6). Others had more positive experiences, typified by line managers and colleagues ‘checking in’ with them regularly and not feeling ‘rushed back’.

There were mixed experiences of contact with OH services, with personnel often viewed as well-meaning, but little available in terms of advice, referrals and workplace adjustments. Those working spoke about delays in accessing appropriate and timely referrals to and support from OH (Quote 5, Table 6), a lack of knowledge around LC amongst OH personnel (particularly early in the pandemic) and/or a lack of support for reasonable adjustments. Those off-work perceived little support available from OH for addressing symptoms or until they were ready to return to work (Quotes 6 and 7, Table 6). For some, OH recommendations around specific measures (maintaining contact, reducing hours) were not always followed by line managers due to pressures faced by the team (and wider NHS). For a small number, OH helped put plans and support in place, for workplace adjustments, to aid return to work (Quote 8, Table 6). OH had also facilitated access to services including occupational therapy and physiotherapy. Given their occupational exposure to COVID-19 and their continued service during the pandemic, many resented the lack of recognition of their LC or support to return or remain at work (Quote 9, Table 6).

Of the 13 individuals off-work, 12 continued to be paid their salary through the UK Government LC special leave fund. There was, however, uncertainty and anxiety around how long this would continue with little official communication (Quote 10, Table 6). The other (a nurse), disappointed by the lack of support offered by her workplace, took early retirement. Some working reduced hours also spoke about continuing to be paid their full salary (as above). Four described stresses experienced because of their initial ineligibility, never having had a positive COVID test (Quote 11, Table 6). There were occupational differences with the GPs not provided with the same financial protections as other NHS colleagues due to their independent contractor status. A few nurses were ‘worse off’ as unable to work ‘bank’ shifts they had previously.

## DISCUSSION

This mixed-methods study explored experiences of HCWs self-reporting symptoms of LC. Most participants continued to work but managed complex and dynamic symptoms, with periods of improvement and exacerbation, which were often poorly recognized or understood. Physical health and mental well-being were lower compared to the general population and levels of fatigue were high. Work was often disrupted; participants engaged in a range of strategies: rest, pacing, planning and prioritizing, with work prioritized overall. Overall satisfaction with support from their employer for their LC symptoms was not high, although there were helpful occupational/work-related supports identified. These included being believed about the impact of LC symptoms (particularly fatigue and brain fog) and workplace adjustments that are flexible in line with typically fluctuating symptoms (phased returns, changes to role responsibilities or working hours, flexible working and working at home where feasible, as well as appropriate and timely referrals to OH). Lack of knowledge and understanding of LC, as well as post-pandemic pressures and demands on the NHS and a staffing crisis constrained the support and adjustments offered to some.

A range of NHS workers responded to the survey. Most (93%) were female which is a higher proportion than in NHS Scotland (79% of staff). Nurses and midwives comprised 48% of respondents to the survey (42% of the workforce) and the most common age category was 36–55 years, which compares with the median age of 45 years of the overall workforce [12].

Over 80% of participants reported limitations with day-to-day activities due to LC, with 31% ‘limited a lot’. Data from the Office for National Statistics report 76% and 18%, respectively, for activity limitations [4]. Most reported multiple, dynamic symptoms with periods of improvement and exacerbation. Overall health and HRQL (both physical and mental components) were rated poorer by participants than in other general population surveys [25] although these were similar to another study of the long-term effects of COVID-19 on health and social services workers [14]. The mean EQ-5D-VAS score was 61.5 which is lower (indicating poorer HRQL) than reported in a large general population data linkage study of laboratory-confirmed COVID-19 infections [26], and the PHOS-COVID study of a hospitalized cohort [27] and reflects data reported from a systematic review [28].

Almost a fifth of HCWs reported they were unable to work due to health problems associated with LC and most had attempted to return to work (some on multiple occasions). Returning to work too soon was detrimental and often exacerbated symptoms, necessitating further time off. Some felt pressured to return or guilty at being off and pressures on NHS and colleagues was often a root cause of this. From an OH perspective, measures to promote sustained return should include a phased return longer than the typical 4–6 weeks, and regular managerial review and adjustment to consider the relapsing–remitting nature of symptoms [29].

The sample size (*n* = 50) is large for qualitative work and enabled us to capture a range of experiences and voices of HCWs. The interviews were in-depth and lengthy, as often the first-opportunity participants had to share their experience in entirety. Given their debilitating symptoms, this was a commitment which may have prevented them from engaging in other activities for hours or days. These interviews were cathartic but also fuelled by their desire to improve LC experiences and outcomes through sharing their experiences.

There were differences in experiences of workplace support across occupational groups, work context and NHS Boards. There was little mention or discussion of OH amongst those working in primary health care (3/12 participants). Where helpful, this had been characterized by understanding and signposting/ access to other services (e.g. LC physiotherapist). Few medics discussed contact with OH, where mentioned, this was seen as unhelpful in meeting their needs in returning to work. GPs’ overall experience was determined by the level of understanding and support from their GP ‘partner’ colleagues. In contrast, more nurses had OH contact and more positive experiences. As well as a nurse who had taken early retirement, two others (AHP and medic in senior positions) were considering early retirement due to the impact their symptoms had on their ability to fulfil their roles.

Self-management strategies prioritizing work above other aspects and time off were reported. Having help with childcare and domestic chores from family was helpful, allowing rest in preparation for work. Additionally, checking over work to avoid mistakes or getting others to do this was discussed, particularly by those with cognitive symptoms. Participants (particularly medics) reported previous strategies of ‘pushing through’ illness to continue at work had been detrimental to their health with LC.

Regarding limitations, survey respondents were recruited through social media; self-selecting participation, so verifying NHS employment was not possible. As of 2023, the number of NHS workers in Scotland is reported as 156 216 [30]; this survey sampling only a small percentage of this total. Given recruitment methods, participants sampled may not be the most unwell with LC. Participants were predominantly nurses, female and White; broadly reflecting socio-demographic characteristics of NHS workers in Scotland [12]. Maximum variation sampling for interviews attempted to redress imbalances in socio-demographic and occupation characteristics. Future research should explore the experiences of LC in under-represented groups including lower socioeconomic groups and ethnic minorities. There was no control group to compare response outcomes, although published data available from other cohorts of individuals with LC reported comparable HRQL and symptom profiles. Recall bias potential in this study appeared to be low; many participants still experiencing symptoms of LC at the time of interviews. However, we acknowledge recall bias is a possibility in this study, perspectives shared are dependent on the accuracy of participant recall and reporting of experiences in both survey and interviews.

Our study highlights the prolonged impact of LC symptoms on a range of HCWs, detrimentally affecting both physical and mental well-being and ability to continue working. Due to the variability and dynamic nature of symptoms, workplace support and flexible policies are needed to help keep staff in the NHS.

## Supplementary Material

kqad117_suppl_Supplementary_Table_S1
