# Supplementary material for: Lived experience of work and long COVID in healthcare staff
Source: Occup Med (Lond). 2023 Dec 10;74(1):78–85. doi: 10.1093/occmed/kqad117 (PMC10875925; doi:10.1093/occmed/kqad117)
Supplement: kqad117_suppl_Supplementary_Table_S1 [file kqad117_suppl_supplementary_table_s1.docx]

**Supplementary data**

**Table 1. Long COVID symptoms reported, n (%)**

| Symptoms reported |  |
| --- | --- |
| Fatigue | 412 (88) |
| ‘Brainfog’ | 374 (79) |
| Breathlessness | 324 (69) |
| Sleep disturbance | 254 (54) |
| Heart palpitations | 227 (48) |
| Headache | 220 (47) |
| Joint pain | 216 (46) |
| Muscle pain | 216 (46) |
| Feelings of anxiety | 197 (42) |
| Chest tightness | 195 (41) |
| Post exertional malaise | 169 (36) |
| Loss of taste/smell | 166 (35) |
| Dizziness | 158 (34) |
| Chest pain | 145 (31) |
| Pins and needles; numbness in hands and feet | 139 (30) |
| Cough | 128 (27) |
| Symptoms of depression | 124 (26) |
| Diarrhoea | 94 (20) |
| Nausea | 92 (20) |
| Tinnitus | 91 (19) |
| Sore throat | 81 (17) |
| Loss of appetite | 75 (16) |
| Abdominal pain | 73 (16) |
| Skin rashes | 64 (14) |
| Fever | 63 (13) |
| Earache | 54 (11) |
